# Collective interaction effects associated with mammalian behavioral traits reveal genetic factors connecting fear and hemostasis

**DOI:** 10.1186/s12888-018-1753-4

**Published:** 2018-06-05

**Authors:** Hyung Jun Woo, Jaques Reifman

**Affiliations:** Biotechnology High Performance Computing Software Applications Institute, Telemedicine and Advanced Technology Research Center, U.S. Army Medical Research and Materiel Command, Fort Detrick, MD USA

**Keywords:** Post-traumatic stress disorder, Anxiety, Quantitative trait, Epistasis, Behavioral genetics

## Abstract

**Background:**

Investigation of the genetic architectures that influence the behavioral traits of animals can provide important insights into human neuropsychiatric phenotypes. These traits, however, are often highly polygenic, with individual loci contributing only small effects to the overall association. The polygenicity makes it challenging to explain, for example, the widely observed comorbidity between stress and cardiac disease.

**Methods:**

We present an algorithm for inferring the collective association of a large number of interacting gene variants with a quantitative trait. Using simulated data, we demonstrate that by taking into account the non-uniform distribution of genotypes within a cohort, we can achieve greater power than regression-based methods for high-dimensional inference.

**Results:**

We analyzed genome-wide data sets of outbred mice and pet dogs, and found neurobiological pathways whose associations with behavioral traits arose primarily from interaction effects: γ-carboxylated coagulation factors and downstream neuronal signaling were highly associated with conditioned fear, consistent with our previous finding in human post-traumatic stress disorder (PTSD) data. Prepulse inhibition in mice was associated with serotonin transporter and platelet homeostasis, and noise-induced fear in dogs with hemostasis.

**Conclusions:**

Our findings suggest a novel explanation for the observed comorbidity between PTSD/anxiety and cardiovascular diseases: key coagulation factors modulating hemostasis also regulate synaptic plasticity affecting the learning and extinction of fear.

**Electronic supplementary material:**

The online version of this article (10.1186/s12888-018-1753-4) contains supplementary material, which is available to authorized users.

## Background

Mammalian behavioral traits, such as burrowing and parenting in wild mice [1, 2], as well as emotional behaviors in lab mice [3], domesticated animals [3, 4], and pets [5, 6], have strong genetic components that interact tightly with the environment. Animal models, which allow transgenic experiments and controlled phenotyping, also help us understand the neurobiological bases of human psychiatric disorders, such as schizophrenia, autism, depression, anxiety, and post-traumatic stress disorder (PTSD). Recent developments in genome-wide association studies have made it possible to perform unbiased, high-resolution interrogation of associated loci. However, such studies have largely been limited to human genetics, in which typical linkage disequilibrium (LD) between individuals is relatively small and high-quality reference panels of common variants are available [7].

Recent studies of outbred mouse stocks [8, 9] with lower degrees of relatedness than lab mice and of multiple breeds of domesticated animals [5, 6] have demonstrated the feasibility of genome-wide mapping of behavioral traits. Although near gene-level mapping resolution has been achieved, levels of LD between variants in many associated loci remain substantially higher than in typical human studies. With higher overall LD, difficulties in interpreting the results of association tests, in which each single-nucleotide polymorphism (SNP) is treated separately, are more pronounced than in human samples. In this context, analytical approaches that test groups of variants for collective association with traits have the potential to reveal hidden genetic factors otherwise undiscoverable by analyzing independent loci.

Here, we report the development of an analytical method to infer collective genetic associations of a group of SNPs with quantitative traits. In this method, which is analogous to a similar approach for binary case-control phenotypes [10–12], pre-selected groups of variants (e.g., genes or pathways) are tested for their association with phenotypes while taking into account the distributions of both genotypes and phenotypes within the cohort.

In conventional association studies, loci highly associated with quantitative traits are first identified using linear regression-based methods, and the putative causal genes near the loci are tested for enrichment in curated databases of pathways. Studies targeting epistatic effects have largely focused on extending single-SNP methods, namely via exhaustive or selective testing of SNP pairs [13–15]. Statistical models considering a large number of variants necessarily require regularization or variable selection. Such regularized high-dimensional inferences have a wide range of applications, including inference of gene expression network structures [16]. More specifically, in the context of quantitative trait analysis, such examples include studies aiming to build genomic predictors that employ aggregates of non-interacting, genome-wide SNPs [17]. In our approach for quantitative trait association analysis, we first select all variants proximal to genes belonging to a given pathway, and infer the collective association strength of these variants while taking into account the aggregated effect of interactions between them. This inference goes beyond linear regression and its multi-dimensional extension, ridge regression (RR), by accommodating nonuniform genotype distributions. The high degree of polygenicity observed in human psychiatric disorders [18], as well as evidence that prioritizing variants based on functional annotations enhances power [19, 20], suggests that collective genetic effects uniquely considered in the proposed method potentially make similarly important contributions to mammalian behavioral traits.

We first used simulated data to demonstrate that substantially higher power could be achieved by such collective inference than by independent loci inference of quantitative trait associations and RR-based multi-locus tests. We chose RR for comparison because it extends linear regression―the main approach used for most association tests using single variant data―in a manner analogous to how our approach uses penalizer-based regularization. We then applied our method to the recent data sets of behavioral traits in outbred mice [8] and dogs [5], analyzing five behavioral assays for mice (fear conditioning, prepulse inhibition, elevated plus maze, forced swim test, and sleep) and fear-related personality traits for dogs. We tested the association of SNP groups formed by curated pathways, while including interaction effects within each SNP group. The classes of biological processes represented by highly ranked pathways associated with each trait, together with known experimental evidence from the literature, provide a markedly enhanced understanding of key psychiatric conditions, including fear, anxiety, and depression [21]. In particular, our inference results for fear conditioning suggest that γ-carboxylated proteases (thrombin and other coagulation factors) play a central role in modulating fear, consistent with recent experimental findings [22, 23], and offer a possible explanation for the comorbidity of PTSD in blood pressure [24] as well as coronary diseases [25, 26]. Our results from dog personality trait data reported by Ilska et al. [5]—fear of noise and fear of humans/objects—provided further support to this main conclusion.

## Methods

### Continuous discriminant analysis for quantitative traits

We formulated and implemented a collective inference algorithm adapted to genotype-quantitative trait data sets, as described in this subsection. We denote the data as *D* = {**a**^*k*^, *y*_*k*_}, where *n* is the total number of individuals and *k* = 1, …, *n*; **a**^*k*^ denotes the genotype count vector for individual *k* (components ${a}_i^k=0,1,2$ and *i* = 1, …, *m*, where *m* is the number of SNPs); and *y*_*k*_ is a continuous-variable phenotype of individual *k*. The log-likelihood of a statistical model is defined as:

1 $$ L=\sum \limits_k\ln \Pr \left({\mathbf{a}}^k,{y}_k\right)=\sum \limits_k\ln \Pr \left({\mathbf{a}}^k|{y}_k\right)+A, $$

where *A* = ∑_*k*_ ln Pr(*y*_*k*_) is the likelihood of the marginal distribution of the phenotype. We assume that the latter is distributed normally with mean *μ* and variance *σ*^*2*^. Maximizing *L* with respect to these two parameters leads to their estimates $\widehat{\mu}$ and ${\widehat{\sigma}}^2$ (the sample mean and variance), which complete the specification of the marginal phenotype distribution. The conditional genotype distribution is modeled as:

2 $$ \Pr \left(\mathbf{a}|y\right)=\frac{e^{H\left(\mathbf{a};y\right)}}{Z(y)}, $$

where

3 $$ H\left(\mathbf{a};y\right)=\sum \limits_{l=0}^1{y}^l\left[\sum \limits_i{h}_i^{(l)}\left({a}_i\right)+\sum \limits_{i<j}{J}_{ij}^{(l)}\Big({a}_i,{a}_j\Big)\right] $$

and $Z(y)=\sum \limits_{\mathbf{a}}{e}^{H\left(\mathbf{a};y\right)}$ is the normalization factor. In Eq. (3), the two terms inside the brackets represent single-SNP and interaction effects, respectively, with parameters $\theta =\left\{{h}_i^{(l)}(a),{J}_{ij}^{(l)}\left(a,b\right)\right\}$ defined for SNP indices *i*, *j* = 1, …, *m*, genotype indices *a*, *b* = 0, 1, 2, and the index representing phenotype-independent and -dependent effects, *l* = 0, 1. These parameters are set to zero if *a* = 0 or *b* = 0. The null hypothesis (no association between genotype *a*_*i*_ and phenotype *y*) is then represented by the condition ${h}_i^{(1)}(a)={J}_{ij}^{(1)}\left(a,b\right)=0,$ under which Eq. (2) becomes independent of *y*. Maximization of *L* in Eq. (1) with respect to these parameters involves computing derivatives:

4a $$ \frac{\partial L/n}{\partial {h}_i^{(l)}(a)}={\widehat{f}}_i^{(l)}(a)-{f}_i^{(l)}(a)-{\lambda}_1{h}_i^{(l)}(a), $$

4b $$ \frac{\partial L/n}{\partial {J}_{ij}^{(l)}\left(a,b\right)}={\widehat{f}}_{ij}^{(l)}\left(a,b\right)-{f}_{ij}^{(l)}\left(a,b\right)-{\lambda}_2{J}_{ij}^{(l)}\left(a,b\right), $$

where ${\widehat{f}}_i^{(l)}(a)={n}^{-1}{\sum}_k{y}_k^l\delta \left({a}_i^k,a\right)$ and ${\widehat{f}}_{ij}^{(l)}\left(a,b\right)={n}^{-1}{\sum}_k{y}_k^l\delta \left({a}_i^k,a\right)\;\delta \left({a}_j^k,b\right)$ are the (phenotype-weighted if *l* = 1) sample genotype frequency and covariance, respectively; the Kronecker delta symbol is defined as *δ*(*a*, *b*) = 1 if *a* = *b* and 0 otherwise; and the corresponding quantities without the hat are population averages defined by:

5a $$ {f}_i^{(l)}(a)=\frac{1}{n}\sum \limits_k{y}_k^l\sum \limits_{\mathbf{a}}\delta \left({a}_i,a\right)\Pr \left(\mathbf{a}|{y}_k\right), $$

5b $$ {f}_{ij}^{(l)}\left(a,b\right)=\frac{1}{n}{\sum \limits}_k{y}_k^l\sum \limits_a\delta \left({a}_i,a\right)\delta \left({a}_j,a\right)\Pr \left(a|{y}_k\right). $$

The aforementioned convention of setting the parameters to zero whenever *a* or *b* is zero makes the total number of unknown parameters equal to that for the sample genotype frequencies and covariances after taking into account constraints associated with their normalization conditions [10]. In Eq. (4), the last terms penalize overfitting under small sample sizes by forcing single-SNP and interaction parameters to be close to 0. The penalizers *λ*_1_ and *λ*_2_ are determined below by cross-validation.

To calculate Eq. (2) and use it for Eqs. (4) and (5), an approximate treatment is necessary. We used the pseudo-likelihood (PL) method [27], which replaces the full distribution by a product over single-SNP distributions conditional on the data:

6 $$ \Pr \left(\mathbf{a}|{y}_k\right)\approx \prod \limits_i\Pr \left({a}_i|{a}_{j\ne i}^k,{y}_k\right)=\prod \limits_i\frac{e^{H_i\left({a}_i|{\mathbf{a}}^k;{y}_k\right)}}{\sum_b{e}^{H_i\left(b|{\mathbf{a}}^k;{y}_k\right)}}, $$

where

7 $$ {H}_i\left(a|{\mathbf{a}}^k;{y}_k\right)=\sum \limits_{l=0}^1{y}_k^l\left[\sum \limits_i{h}_i^{(l)}(a)+\sum \limits_{j\ne i}{J}_{ij}^{(l)}\Big(a,{a}_j^k\Big)\right]. $$

**Fig. 1:**
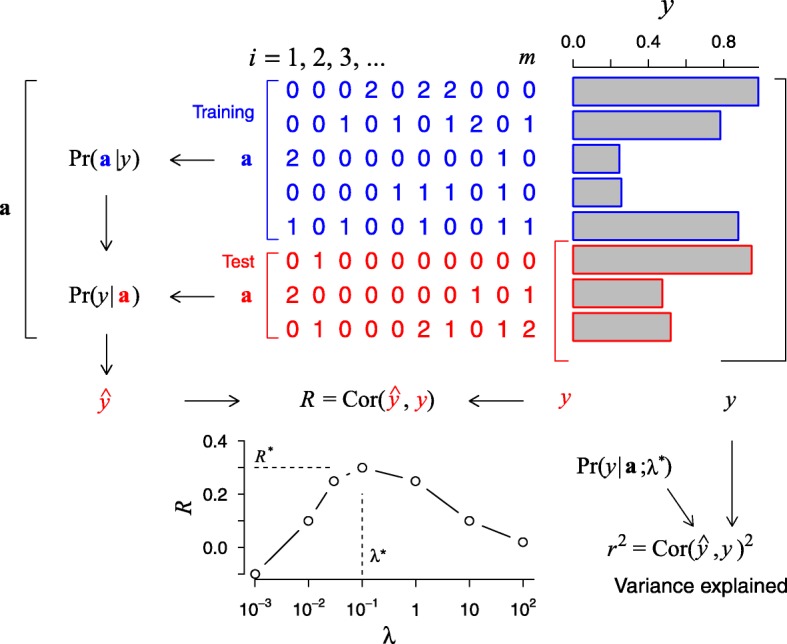
Continuous discriminant analysis for quantitative traits. Paired genotype (**a**)-phenotype (*y*) data for individuals are divided into training and test sets. The training set is used to model the conditional distribution Pr(**a**|*y*), while including the interaction effects between all *m* SNPs. Parameters with large magnitudes that often result from insufficient data are made unfavorable by the penalizer λ. Bayes’ rule is then used to obtain Pr(*y*|**a**) and applied to predict phenotype values for individuals in the test group. The correlation *R* between the predicted and actual phenotypes is optimized with respect to λ. Because of the training/test set division, *R*^*2*^ is in general not equal to *r*^2^, the proportion of phenotype variance explained by genetic predictors. The latter can be estimated by using the optimized penalizer and repeating the inference

The use of Eqs. (6) and (7) in Eq. (5) allows one to avoid full marginalization over genotypes via the use of single-SNP densities conditional on the data, making the computation tractable for large numbers of interacting SNPs. We determined the penalizers *λ*_1_ and *λ*_2_ by optimizing the Bayes estimator for phenotypes

8 $$ \overline{y}\left(\mathbf{a}\right)=\int y\Pr \left(y|\mathbf{a}\right) dy=\frac{\int y\Pr \left(\mathbf{a}|y\right)\Pr (y) dy}{\int \Pr \left(\mathbf{a}|y\right)\Pr (y) dy} $$

evaluated by the trapezoidal rule under cross-validation, in which we divided the *n* individuals into training and test groups at a 4:1 ratio, inferred parameters from the training group under the given penalizers, and calculated Eq. (8) for the test group individuals (Fig. 1). We selected *λ*_1_ and *λ*_2_ that maximized the prediction score, defined as the correlation between predicted and actual phenotype values, $R=\mathrm{Cor}\left[{y}_k,\overline{y}\left({\mathbf{a}}^k\right)\right]$.

The software GeDI (Genotype distribution-based inference), which implements the quantitative trait analysis algorithm, is available at https://github.com/BHSAI/GeDI.

### Ridge regression

For purposes of comparison, we implemented RR, which fits the data to the model

9 $$ y\left(\mathbf{a}\right)=\alpha +\sum \limits_i{a}_i{\beta}_i+\sum \limits_{i<j}{a}_i{a}_j{\gamma}_{ij}+\varepsilon, $$

where $\varepsilon \sim \mathrm{N}\left(0,{\sigma}_y^2\right)$, or $\overline{\mathbf{y}}=\mathbf{X}\kern0.1em \mathbf{b}$, where $\overline{\mathbf{y}}$ is the column vector with elements *y*_*k*_, **b** is the coefficient vector with *p =* 1 *+ m + m* (*m –* 1) / 2 elements {1, *β*, *γ*}, and **X** is the *n* × *p* data matrix with 1 for the first column, ${a}_i^k$ for columns 2 to *m* + 1, and ${a}_i^k{a}_j^k$ for the rest. This approach can be regarded as approximating the log likelihood of the data as:

10 $$ L=\sum \limits_k\ln \left[\Pr \left({y}_k|{\mathbf{a}}^k\right)\Pr \left({\mathbf{a}}^k\right)\right]\approx \sum \limits_k\ln \Pr \left({y}_k|{\mathbf{a}}^k\right)=-\frac{1}{2{\sigma}_y^2}\sum \limits_k{\left[{y}_k-y\left({\mathbf{a}}^k\right)\right]}^2-\frac{n}{2}\ln \left(2{\pi \sigma}_y^2\right), $$

where the marginal genotype distribution is assumed to be uniform. We added a penalizer and maximized *L* − *λ*^′^ **b**^*t*^**b** to obtain:

11 $$ \widehat{\mathbf{b}}={\left({\mathbf{X}}^t\mathbf{X}+\lambda \mathbf{I}\right)}^{-1}{\mathbf{X}}^t\mathbf{y}=\mathbf{V}{\left({\mathbf{R}}^t\mathbf{R}+\lambda \mathbf{I}\right)}^{-1}{\mathbf{R}}^t\mathbf{y}, $$

where ${\widehat{\sigma}}_y^2={\left(\mathbf{y}-\mathbf{Xb}\right)}^t\left(\mathbf{y}-\mathbf{Xb}\right)/n$, $\lambda ={\lambda}^{\prime }{\widehat{\sigma}}_y^2$, **I** is an identity matrix of dimension *p*, and **V** is a *p* × *n* orthogonal matrix from singular value decomposition [28] of **X**. The second form of Eq. (11) reduces computational costs for *p > n*. To enable this form, we chose *λ* to be uniform for all components of **b** in RR, whereas in CDA we used two distinct penalizers for the single-SNP and interaction terms, respectively.

### Simulated data

We generated simulated data by first randomly assigning parameter values from normal distributions for a given number *m* of interacting SNPs. Phenotype values for a varying number of individuals (sample size) were sampled from the standard normal distribution. We then used Eq. (2) to calculate the probabilities of all possible genotypes (2^*m*^ in total) for each value of *y*_*k*_ and chose one genotype for each individual based on these probabilities. We then applied CDA and RR, performed 5-fold cross-validation, and determined the penalizers by maximizing *R*. For these simulations, we used the dominant model to reduce the computational cost of enumerating all possible genotypes. For all other computations using animal trait data, we used the genotypic model, which includes the dominant model as a special case and generally enhances the power to infer associations relative to the dominant model [12].

### Outbred mice data

We used the genotype data for 1934 mice reported by Nicod et al. [8] and selected animals for which the trait values under consideration were available. The sample size for mice ranged from 1065 to 1716 (Additional file 1: Table S1). We used the corrected mean time freezing during the cue and context tests for fear conditioning, average pulse reactivity for prepulse inhibition, fraction of time in open arms for the elevated plus maze, and sleep length in 24 h, as well as the difference in light and dark periods for sleep (Additional file 1: Table S1). Non-integral dosage values for imputed SNPs were rounded off to integral allele counts. The total number of SNPs for all data sets was 359,559. Fractional trait values between 0 and 1 were log-transformed before use with small constants added in the argument to avoid singularities. We examined the quantile-quantile plots of independent-SNP *p*-values obtained from linear regression for each data set, and sought to eliminate any inflation by stratifying the data set into two sub-samples based on a covariate. The division into male and female mice proved adequate for many traits, whereas forced swim and sleep difference between light and dark periods required different choices (immobility during the first 2 min and average body weight, respectively; Additional file 1: Table S1).

### Meta-analysis

We implemented and used a meta-analysis scheme for collective inference involving multiple sub-samples [12, 29] (two in this work). Each sample was first divided into training and test sets, and the training sets were used to infer single-SNP and interaction parameters separately for each sub-sample. These models were then averaged with sample-size weighting and subsequently used to predict phenotypes for the aggregated test animals of all sub-samples. The prediction score *R* was then optimized with respect to the penalizers.

### Labrador retrievers

We used the genotype data for 885 Labrador Retrievers, reported by Ilska et al. [5]. For the two traits we considered (fear of noise and of humans/objects), the sample sizes were 868 and 882, respectively (Additional file 1: Table S1). The values on the questionnaire scale (from 1 to 5) were log-transformed before use as for mice. We used the first principal component to stratify animals into two groups (large and small principal component values; see Additional file 1: Figure S5) and performed meta-analyses. We chose 110,419 SNPs with known CanFam3 positions [30] for analysis.

### Association testing of pathway-based variant groups

We used mouse and dog pathways from the Reactome database [31] (downloaded on December 23, 2016). For each gene set (mouse/dog orthologs of the human genes in the corresponding human pathway), we formed a union of all SNPs whose positions in the genome were within 50 kb of the coding regions of all genes. We considered all pathways with 5 or more SNPs (1502 and 1459 in total for mice and dogs, respectively). The mouse data set typically contained groups of neighboring SNPs with near perfect LD; before association testing, we used PLINK [32] (window size 50 bp shifted by 5 SNPs, LD threshold 0.9) to prune the SNP set of a given pathway, and then stratified the set into two covariate-dependent subgroups and performed collective inference meta-analysis. We chose this pruning procedure on the basis of our previous work showing that pathway-based association tests are insensitive to local LD, typically from 1.0 to ~ 0.5 [10, 12]. Pruning with a threshold of 0.9 substantially reduced the number of SNPs for each pathway in the mouse data set, allowing for consideration of much larger pathways than without pruning. We used the dog data set without pruning because it did not contain large chunks of SNPs with maximal LD.

The main statistic we used for association testing was the prediction score *R*, defined as the correlation between the predicted and actual phenotypes calculated for individuals in the test set within cross-validation. The use of cross-validation allows us to avoid any bias arising from overfitting. We optimized the prediction score *R* with respect to λ_1_ and λ_2_, which we allowed to vary independently between 0.01 and 100. We included in this optimization the special case in which the interactions were turned off (λ_2_ = ∞).

To estimate the *p*-values of SNP sets, we used the fact that our main statistic is a correlation, for which the null distribution is well-known analytically. We used *P* = 1 − Φ  (*z*), where Φ is the cumulative distribution function of the standard normal distribution, $z=\sqrt{n-3}\left(f-{f}_0\right)$, where *f* = (1/2) ln[(1 + *R*)/(1 − *R*)] and *f*_0_ = (1/2) ln[(1 + *R*_0_)/(1 − *R*_0_)] (Fisher’s transformation). To obtain the mean correlation *R*_0_ under the null hypothesis necessary in this formula, we repeated the inference 10 times with phenotype labels permuted and calculated the mean of the correlations. This mean value was typically close to zero and positive but negative for some pathways. We tested this null distribution (the Fisher-transformed correlation is normally distributed) for a selection of SNP sets and calculated *p*-values by phenotype-label permutation directly as the fraction of replicates among ~ 1000 for which *R* < *R*_0_ (Additional file 1: Figure S3). We also tested the possibility of assuming *R*_0_ = 0, and found it to yield substantial deviations from the diagonal in the quantile-quantile plots for pathways with *P* close to 1; the only choice that produced correct *p*-value distributions was to allow *R*_0_ to be negative and to estimate it by phenotype permutation for each pathway (Additional file 1: Figure S4 and Fig. 4).

We also compared the results based on the main Reactome pathway set with those based on an updated version (February 2018). We tested the association of 102 pathways that had been added to the main database with fear conditioning (cued test) and found that the highest ranked pathways had *P* values on the order of ~ 10^− 3^ (Additional file 1: Table S2).

### Heritability

We estimated the broad-sense heritability of a pathway as follows: for a given pathway, we first divided the cohort into two halves and used the first half to identify the optimal values of penalizers λ_1_ and λ_2_, which maximize the prediction score *R* under cross-validation. We then applied the inference for the second half under these penalizer values and calculated the squared correlation between the predicted and actual trait values. For comparison, we used the software GCTA [33] and LDAK [34] to estimate the proportion of variance explained by non-interacting SNPs (narrow-sense heritability) contained in the same pathway (Fig. 8). In the latter calculations, we included sex (cued fear and prepulse inhibition) and the first principal component (fear of noise in dogs) as covariates.

## Results

### Collective inference for quantitative traits

We implemented the algorithm we termed the continuous discriminant analysis (CDA) procedure for quantitative traits, where the genotype-phenotype data for *m* SNPs and *n* individuals were fit to a joint distribution model using the maximum likelihood method (Fig. 1). We first modeled the phenotype data by a normal distribution. We then considered the genotype distribution conditional on phenotypes parameterized by the sum of the additive and interaction terms of all variants. For binary phenotypes, these additive and interaction parameters are defined separately for case and control groups [12]. In contrast, for a single cohort with quantitative traits, we considered these parameters to be linear functions of the phenotype value. The intercept and slope of the additive and interaction parameters of the genotype distribution conditional on phenotypes were then inferred using the maximum likelihood method (see Methods). For the typical model sizes we considered (*m*, the number of SNPs, of up to ~ 1000), the total number of model parameters including the interaction terms often greatly exceeded the sample sizes, and regularization was necessary to prevent overfitting. We adopted a cross-validation scheme in which we divided the sample into training and test groups, and performed inference by using only training individuals (Fig. 1) in the presence of penalizers. The genotype distribution conditional on phenotypes was then used to predict the phenotype values for test individuals. We calculated the correlation *R* between the true and predicted phenotypes as the performance measure and maximized it with respect to the penalizers to determine the optimal fit. Because of the training/test group division, this prediction score *R*^2^ is distinct from the usual proportion of variance explained by regression (*r*^2^). We estimated the latter by dividing the sample into two parts, using the first half to determine the optimal penalizer values, and using the second half to calculate the squared correlation from self-prediction.

**Fig. 2:**
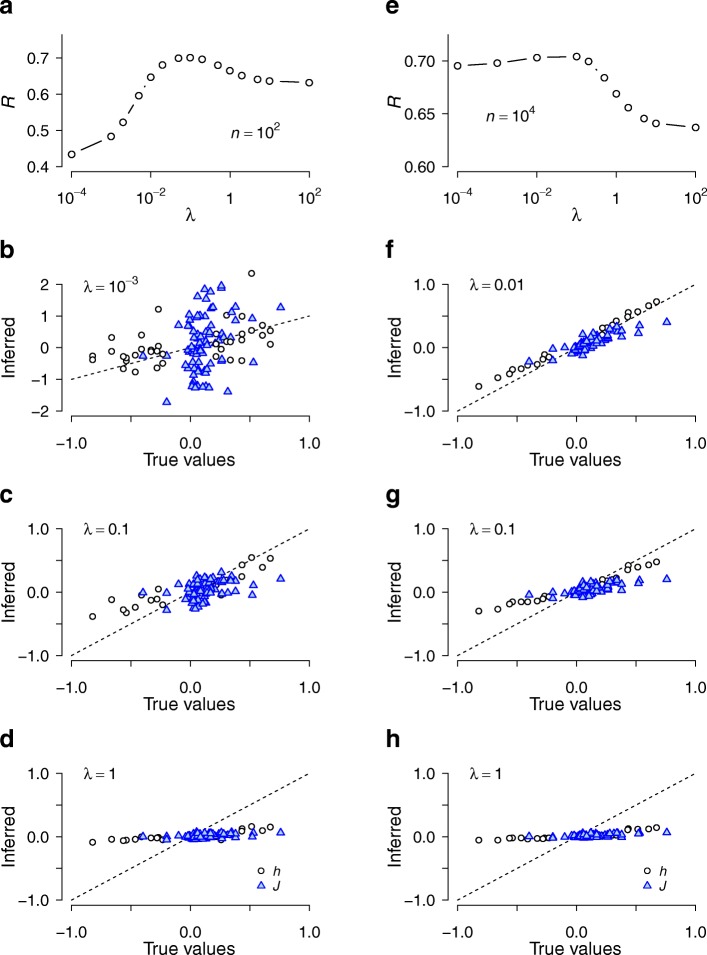
Regularized inference of genotype-quantitative trait associations for two different sample sizes and varying penalizer values. **a–d** Simulated data with *n* = 100, where the overall dependence of prediction score *R* (correlation between predicted and actual phenotype values for test individuals) is shown in **a**, and **b–d** show the comparisons between predicted and true parameter values (single-SNP parameter *h* and interaction *J* for each SNP and SNP pairs, respectively) for three different penalizer λ values. Closer to the diagonal is better. Note that the condition λ = 0.1 in **c** optimizing *R* (see **a**) gives the best fit. **e–g** Analogous results for *n* = 10^4^. The sample size is large enough such that overfitting under small λ is negligible. The number of SNPs was *m* = 5 and the dominant model was used. Parameters were generated randomly from normal distributions: *h*^(0)^ ~ N(−0.3, 0.1^2^), *h*^(1)^~ N(0.3, 0.1^2^), *J*^(0)^ ~ N(0, 0.05^2^), and *J*^(1)^ ~ N(0.1, 0.05^2^). The phenotype values {*y*_*k*_} for *k* = 1, …, *n* were generated from N(0, 1) and, for each individual, the conditional genotype distribution given by Eqs. (2–3) was used to generate genotypes

**Fig. 3:**
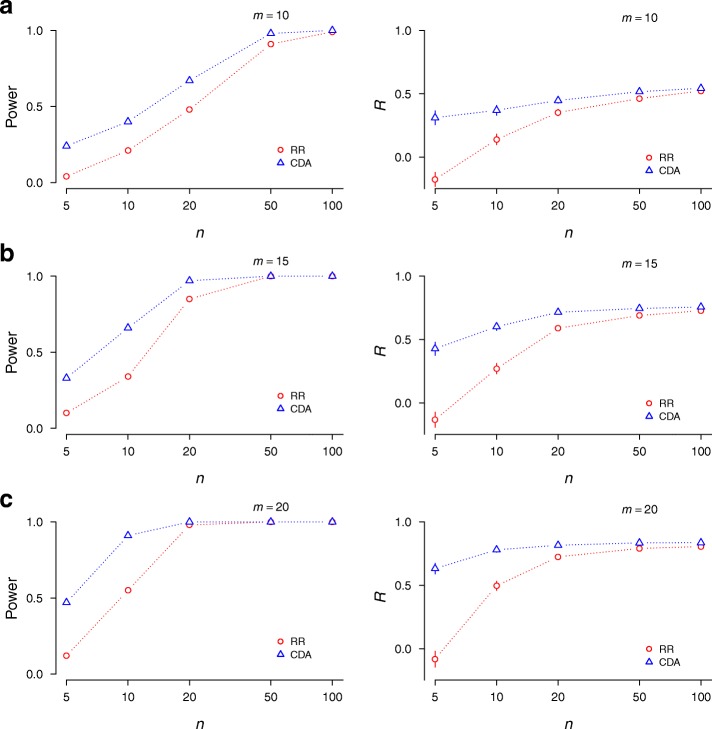
Collective inference performance. Ridge regression (RR) and CDA were compared using simulated data. We first sampled phenotype values of *n* individuals from the standard normal distribution. Restricting ourselves to the number of SNPs (*m* ≤ 20) allowing for the enumeration of all possible genotypes, we then assigned single-SNP and interaction parameters for *m =* 10 (**a**), *m* = 15 (**b**), and *m* = 20 SNPs (**c**) from normal distributions *h*_*i*_^(0)^ ~ N(0, 0.01), *J*_*ij*_^(0)^ ~ N(0, 0.01), *h*_*i*_^(1)^ ~ N(0, 0.01), and *J*_*ij*_^(1)^ ~ N(0.1, 0.01), under the dominant model. We next calculated the genotype distribution conditional on phenotypes for all possible genotypes, and chose a genotype for each individual based on this distribution. We repeated this sampling for 100 replicates. For each data set, we applied RR and CDA collective inference, using a single penalizer λ determined by optimizing *R* by cross-validation (right column). Power was defined as the proportion of replicates for which *P <* 0.05

We tested our algorithm using simulated data, for which *m* was small enough so that we could enumerate all possible genotypes. We maximized the prediction score *R* as a function of the penalizer under conditions where the inferred interaction parameters were closest to the true values for a given sample size (Fig. 2). Cross-validation efficiently identified the regularization conditions that optimized prediction, while avoiding overfitting for small sample sizes. We then compared the power to detect the overall significance of a group of interacting SNPs under CDA and RR [28] (i.e., linear regression, including all interaction terms, and regularized by the same cross-validation scheme as that for CDA). The optimized prediction score *R* was higher for CDA than for RR in all cases; in addition, the differences were greater the smaller the sample size *n* and the larger the number of SNPs *m*, which led to higher power (type I error *α* = 0.05, evaluated over multiple replicates of simulated samples) (Fig. 3). These results suggest that for small sample sizes and high dimensionality (number of interacting variants considered), CDA achieves higher power than regression-based methods to detect collective association strengths of interacting SNPs. The statistical significance of an inference scored by *R* can be evaluated using *p*-values obtained from the null distribution of *R* known for normally distributed data (see Methods).

### Behavioral traits of outbred mice

We applied our algorithm to the genotype-quantitative trait data of outbred mice [8] (Additional file 1: Table S1 and Figure S1). Independent SNP *p*-values from CDA for the special case of single SNPs without interaction effects were numerically close to linear regression outcomes over a wide range of significance levels (Additional file 1: Figure S2). To account for the effects of covariates, such as sex, we used meta-analyses with sample size-weighted averaging of parameters [29], where the sample was sub-divided into two subgroups based on covariate distributions. For each group, we separately performed CDA inference and averaged the inferred parameters over the subgroups. In our inference, the association strength was quantified by the correlation *R*, and the corresponding *p*-value was estimated from the known null distribution of normally distributed data (see Methods). We tested this assumption using a selection of SNP sets and estimating their *p*-values directly by permutation sampling, and found good agreement (Additional file 1: Figure S3), which indicated that the computationally expensive permutation-based testing can be avoided in general. We then clustered SNPs into 1502 groups of varying sizes corresponding to pathways [31] and tested the association of each group with behavioral traits. (See Additional file 2: Table S3 for top-ranked pathway lists of all traits considered.)

#### Fear conditioning

**Fig. 4:**
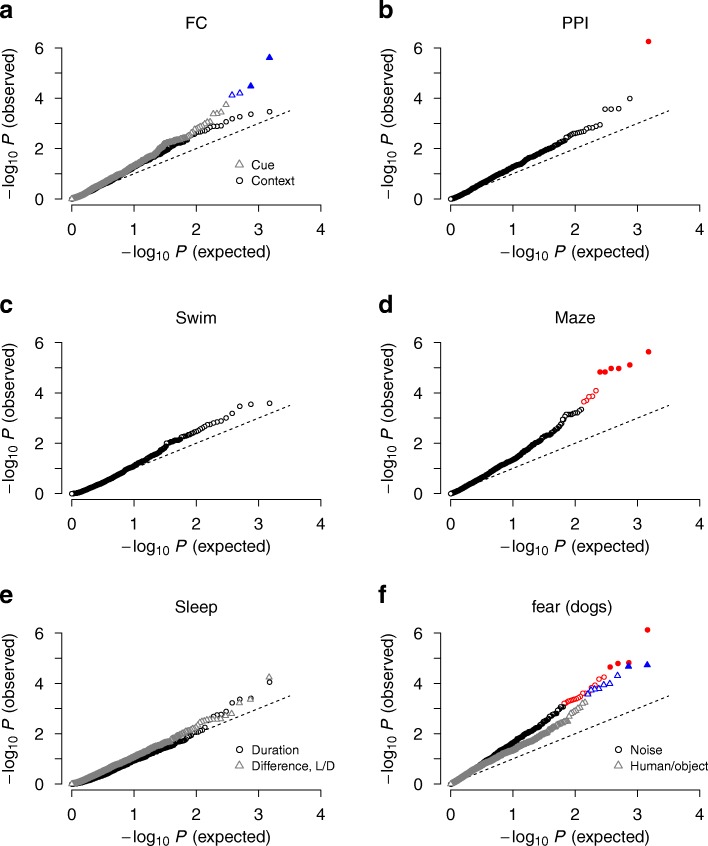
Quantile-quantile plots of behavioral traits for pathway-based SNP groups from CDA inference. **a** Fear conditioning (FC) in cued and context tests. **b** Prepulse inhibition (PPI). **c** Forced swim test. **d** Elevated plus maze. **e** Sleep (total duration and difference in sleep lengths between lighted and dark periods) **f** Fear of noise and humans/objects in dogs. Data sets used are outbred mice (**a–d**) and Labrador Retrievers (**e–f**). Colored symbols and filled symbols represent pathways with false discovery rate < 0.05 and with significance exceeding Bonferroni-corrected threshold, respectively

**Fig. 5:**
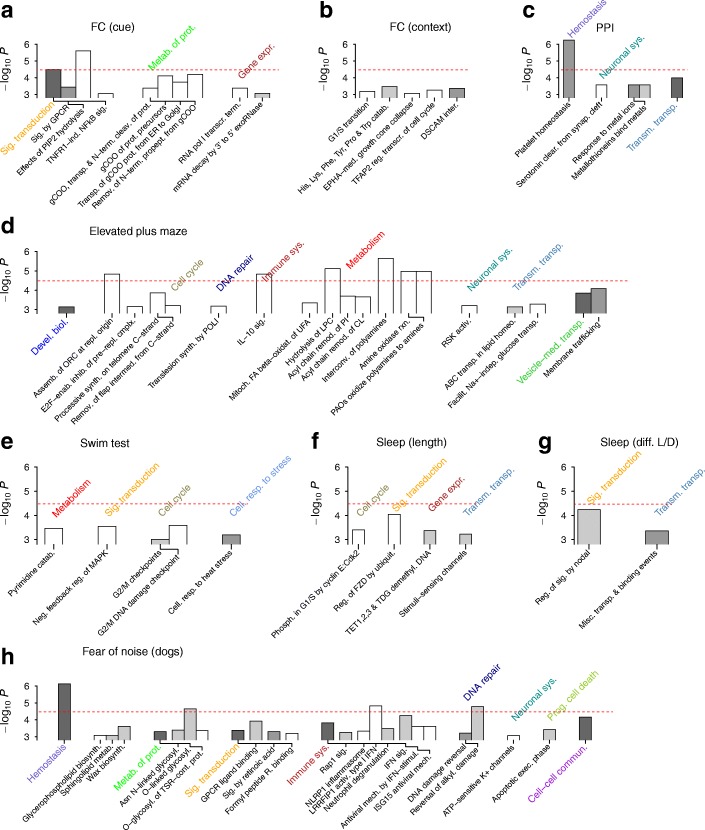
Top-ranked pathways associated with quantitative behavioral traits. Results for mice (**a–g**) and dogs (**h**) are shown. **a** Fear conditioning (FC) cue test. **b** FC context test. **c** Prepulse inhibition (PPI). **d** Elevated plus maze. **e** Forced swim test. **f** Sleep duration in 24 h. **g** Differences in sleep length in light (L) and dark (D) periods. **h** Fear of noise in dogs. Dashed red lines represent Bonferroni-corrected significance thresholds. Groups of pathways belonging to different classes are labeled with colored texts. ABC, ATP-binding cassette; Activ., activation/activates; alkyl., alkylation; assemb., assembly; biol., biology; biosynth., biosynthesis; catab., catabolism; Cdk, cyclin-dependent kinase; cell., cellular; CL, cardiolipin; clear., clearance; cleav., cleavage; cmplx., complex; cont., containing; demethyl., demethylates; devel., development/developmental; DSCAM, Down syndrome cell adhesion molecule; enab., enables; EPH, erythropoietin-producing human hepatocellular receptor; ER, endoplasmic reticulum; exec., execution; expr., expression; FA, fatty acid; facilit., facilitative; FZD, frizzled protein; gCOO, γ-carboxylation/carboxylated; glycosyl., glycosylation; GPCR, G protein-coupled receptor; homeo., homeostasis; IFN, interferon; IL, interleukin; ind., induces/induced; indep., independent; inhib., inhibition/inhibits; inter., interaction; interconv., interconversion; intermed., intermediate; ISG15, interferon-stimulated gene 15; LPC, lysophosphatidylcholine; LRRFIP1, leucine-rich repeat flightless-interacting protein 1; MAPK, mitogen-activated protein kinase; mech., mechanism; med., mediated; metab., metabolism; misc., miscellaneous; neg., negative; NFkB, nuclear factor kappa B; NLRP, NACHT, LRR and PYD domains-containing protein; oxidat., oxidation; PAO, polyamine oxidase; phosph., phosphorylation; PI, phosphatidylinositol; PIP2, phosphatidylinositol phosphate 2; pol I, polymerase I; prog., programmed; propept., propeptide; prot., protein; R., receptor/receptors; Rap, Ras-related protein; reg., regulation/regulates; remod., remodeling; remov., removal; repl., replication; resp., response; RSK, ribosomal 6 kinase; rxn., reaction; sig., signal/signaling; stimul., stimulation; synap., synaptic; synth., synthesis; sys., system; TDG, thymine-DNA glycosylase; term., terminal/terminates/termination; TET, ten-eleven translocation methylcytosine dioxygenase; TFAP, transcription factor activating enhancer binding protein; TNFR, tumor necrosis factor R; transcr., transcription; transm., transmembrane; transp., transports/transporter/transportation; TSR, thrombospondin repeat; ubiquit., ubiquitination; UFA, unsaturated fatty acid

**Fig. 6:**
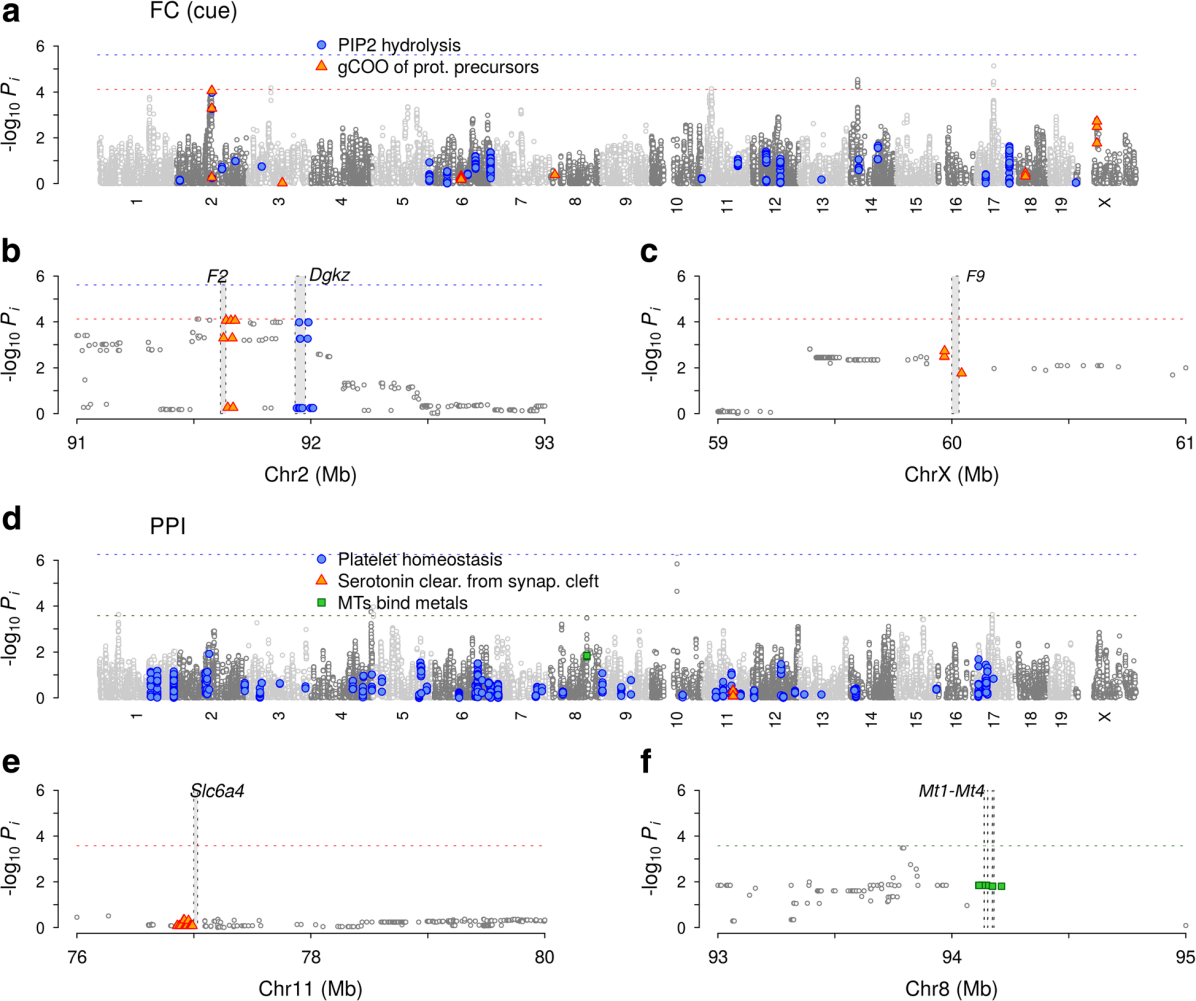
Independent-SNP and collective association levels of variants contributing to pathways. Those highly ranked for mouse behavioral traits are shown. **a** Manhattan plot for fear conditioning, showing single-SNP *p*-values for linear regression. The mouse SNPs for genes in two pathways, *Effects of PIP2 hydrolysis* and *γ-carboxylation of protein precursors* are shown in color. Horizontal lines show the collective inference *p*-values for these two pathways. **b–c** Detailed views of two loci contributing to pathways in **a**. **d–f** Prepulse inhibition and three pathways, *Platelet homeostasis*, *Serotonin clearance from synaptic cleft*, and *Metallothioneins bind metals*. The collective *p*-values of the latter two pathways (bottom horizontal lines) are indistinguishable. Filled rectangles represent the coding regions of genes indicated

We considered two fear conditioning traits: the fraction of time freezing during presentation of a tone (cue test) and that during exposure to the context alone (context test) [8]. Quantile-quantile plots of all pathway gene set-based SNP groups indicated adequate control of genomic inflation under meta-analyses with sex-based subgroups (Fig. 4a). We found stronger associations of top-ranked pathways for the cue test compared to the context test (Fig. 5a–b): for cue testing, the first group of pathways contained two that exceeded the Bonferroni threshold [*Effects of phosphatidylinositol 4,5-phosphate (PIP2) hydrolysis*, *P* = 2.4×10^−6^; *Signal transduction*, *P* = 3.3×10^−5^; see Additional file 1: Figure S4 for *R* values]. The presence of *Signaling by G protein-coupled receptors* (GPCRs; *P* = 3.6×10^−4^), which contains the PIP2 hydrolysis pathway, suggested that the strongest association with cued fear arises from the group of genes involved in post-synaptic signaling by GPCRs during memory consolidation [35]. The PIP2 hydrolysis pathway contained 82 SNPs (after pruning by LD *r*^2^ < 0.9) distributed over ~ 20 genes. None of these individual genomic loci were dominant in association strengths without interaction effects (Fig. 6a), indicating the collective nature of the PIP2 hydrolysis-cued fear association.

The second group of highly ranked pathways contained those involved in γ-carboxylated proteins, including their synthesis, transport in the endoplasmic reticulum (ER) and Golgi apparatus, and modifications (Fig. 5a), of which two had false discovery rates (FDR) < 0.05 (*Removal of N-terminal propeptides from γ-carboxylated proteins*, *P* = 6.3×10^−5^; *γ-carboxylation of protein precursors*, *P* = 7.6×10^−5^). The *γ-carboxylation of protein precursors* pathway contained 10 SNPs after LD pruning near two coagulation factor-coding genes, *F2* (thrombin) and *F9*, in addition to the genes *Bglap*, *Ggcx*, *Gas6*, and *Proc*. Recent studies have revealed that γ-carboxyglutamate-containing coagulation factors, particularly thrombin, in addition to playing central roles in hemostasis of peripheral blood [36], also regulate synaptic plasticity by stimulating protease-activated receptor 1 (PAR1) [22]. PAR1, a GPCR highly expressed on neurons, is activated by the cleavage of its extracellular N-terminus via the action of thrombin [37, 38] as a protease. Coupling of PAR1 to Gα_q_ protein activates phospholipase Cβ (PLCβ), which hydrolyzes PIP2 to generate second messenger molecules, inositol 1,4,5-triphosphate (IP3) and diacylglycerol (DAG), leading to the phosphorylation of cytosolic proteins by protein kinase C and mobilization of Ca^2+^, respectively [37, 39]. Together, the PIP2 hydrolysis and γ-carboxylation pathway groups in Fig. 5a strongly implicate this thrombin-PAR1 signaling pathway in cued fear, and in particular, the dynamic modulation of G protein coupling during long-term potentiation in the amygdala [22]. These results are also consistent with our previous finding of a high association between γ-carboxylation pathways and PTSD in humans [12, 40] (Fig. 4a in Ref. [12]; *Transport of γ-carboxylated protein precursors from ER to Golgi apparatus*, *P* = 9.6×10^−5^ in human PTSD versus *P* = 1.8×10^−4^ in the current study for cued fear).

The third group of pathways associated with cued fear (*P* < 10^−3^) were those for transcription and mRNA decay (*RNA polymerase I transcription termination*, *P* = 4.1×10^−4^; *mRNA decay by 3′ to 5′ exribonuclease*, *P* = 8.7×10^−4^), which are likely relevant in the regulation of synaptic plasticity at the levels of transcription and translation, e.g., by the transport and storage of mRNAs in distal dendrites [41].

Gene sets associated with the amount of freezing during the context test showed a distribution similar to those during the cue test (Fig. 4a) but lacked pronounced groups of highly ranked pathways (Fig. 5b). The highest ranked pathways included those for cell cycle and axon guidance, which likely affect neural development and thereby fear responses.

#### Prepulse inhibition

The inference results for prepulse inhibition (Fig. 5c) were dominated by the top-ranked pathway, *Platelet homeostasis* (*P* = 5.6×10^−7^), whose strong association was clearly collective in nature, containing a large number of variants of individually low association levels scattered across different chromosomes (Fig. 6d). A pathway lower in association strength but nonetheless notable was *Serotonin clearance from the synaptic cleft* (*P* = 2.6×10^−4^), which describes the action of the serotonin transporter encoded by *Slc6a4*, the target gene of numerous antidepressant drugs known as serotonin reuptake inhibitors [42, 43]. SNPs for this pathway consisted solely of those near *Slc6a4*, whose individual association levels were negligible in contrast to their collective *p*-value (Fig. 6e). Serotonin transporter gene variants have previously been linked to startle responses in human subjects [44]. The large body of evidence implicating *Slc6a4* in behavioral traits and psychiatric disorders [42, 43], along with the strong association of *Platelet homeostasis,* suggest that serotonin and its signaling play key roles in prepulse inhibition: in addition to modulating brain functions, serotonin is abundantly stored in platelets outside the brain and regulates vasoconstriction, dilation, and other cardiac functions [45]. A pathway similar in association strength was *Metallothioneins bind metals* (*P* = 2.7×10^− 4^), which contained SNPs near genes *Mt1–4* (Fig. 6f).

#### Elevated plus maze

**Fig. 7:**
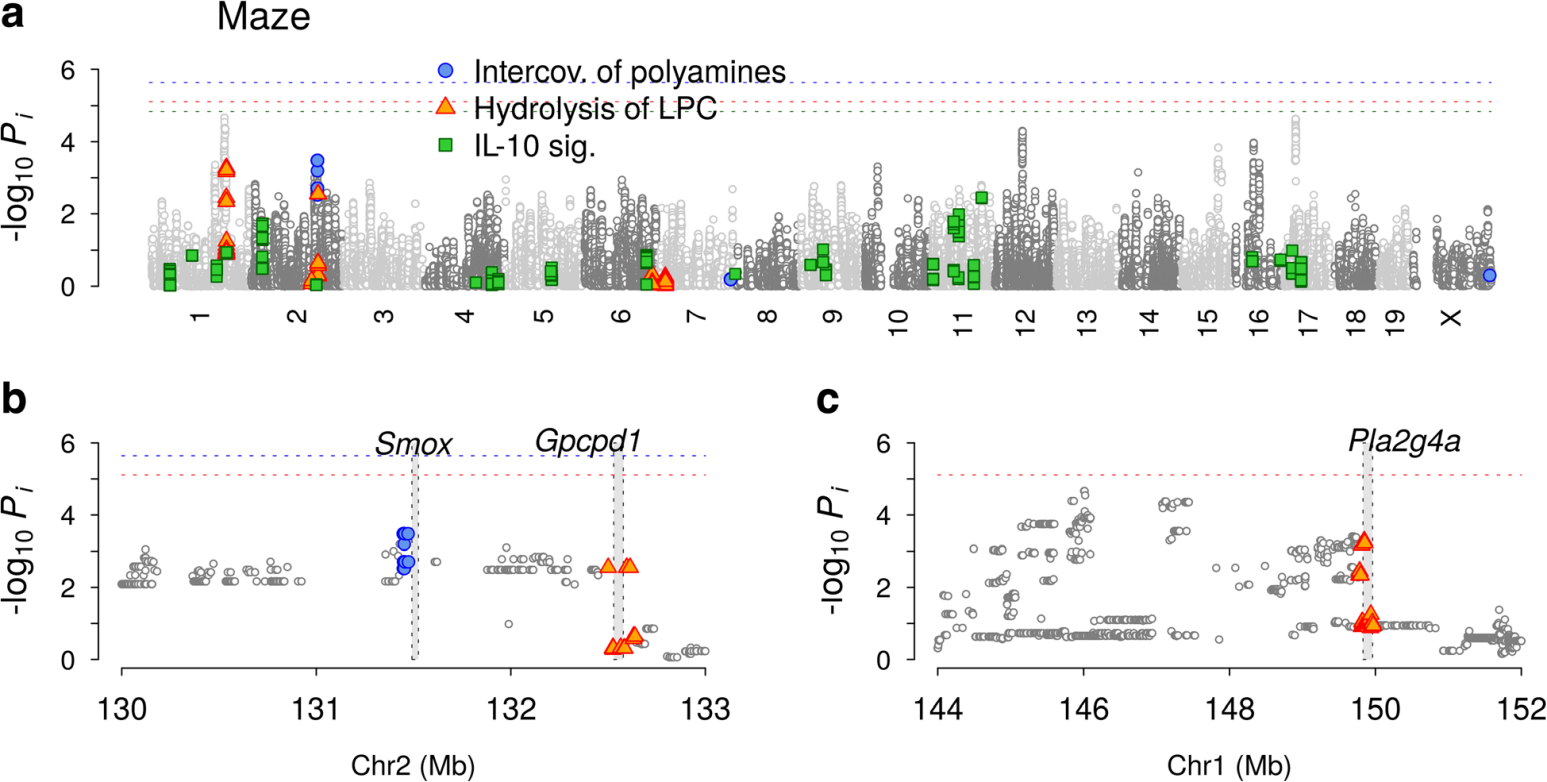
Independent-SNP and collective association levels of variants contributing to pathways associated with elevated plus maze. **a** Manhattan plot and variants in three pathways, *Interconversion of polyamines*, *Hydrolysis of lysophosphatidylcholine*, and *Interleukin-10 signaling*. **b–c** Detailed views of two loci contributing to pathways in **a**. Filled rectangles represent the coding regions of genes indicated

For elevated plus maze test data, we chose the fraction of time spent in closed arms as the trait representing anxiety. A relatively large portion of pathways showed substantial deviations from the null distribution for this trait, while the sex-based meta-analysis still adequately controlled for inflation in pathways (*P* > 0.1) (Fig. 4d). The highest-ranked pathways (Fig. 5d) were *Interconversion of polyamines* (*P* = 2.2×10^−6^), arising from SNPs near the Smox gene (Fig. 7b), and *Hydrolysis of lysophosphatidylcholine* (LPC; *P* = 7.7×10^−6^), containing SNPs near the Pla2g4a and Gpcpd1 genes (Fig. 7b–c). In contrast to these pathways, whose association appeared to arise from SNPs near genes located in one of the loci with strong LD, Interleukin (IL)-10 signaling (*P* = 1.5×10^−5^) was highly polygenic, similar to *Effects of PIP2 hydrolysis* and *Platelet homeostasis* (Fig. 6a,d).

#### Forced swim test and sleep-related traits

Inference for the forced swim test (immobility during the last 4 min as a measure of depression) indicated signs of inflation under sex-based meta-analysis. We performed meta-analyses by sub-dividing the cohort into two groups of high and low immobility during the first 2 min and inferred the association with immobility during the last 4 min separately, so that only the component of depression traits induced by stress (forced swim) would be tested. This choice removed genomic inflation (Fig. 4c) and yielded a top-ranked pathway (Fig. 5e), *Negative feedback regulation of mitogen-activated protein kinase (MAPK) pathway* (*P* = 2.8×10^−4^), along with cell cycle pathways (*G2/M DNA damage checkpoint*, *P* = 2.6×10^−4^). Our finding of the association of MAPK signaling and its negative regulators is consistent with reported evidence for their roles in depression [46, 47]. We additionally tested two sleep-related traits—overall duration and difference in sleep length between light and dark periods [8]—and found *Regulation of Frizzled by ubiquitination* (*P* = 9×10^−5^) of Wnt signaling and *Regulation of signaling by Nodal* (*P* = 5×10^−5^) to be highly associated, respectively, with each trait (Fig. 5f–g).

### Behavioral traits of dogs

To gain further insight into the genetic pathways associated with fear-related traits, we additionally analyzed recent dog personality trait data reported for Labrador Retrievers by Ilska et al. [5]. We chose two dog traits for analysis: fear of noise and of humans/objects. Independent SNP analysis by linear regression indicated substantial inflation from the population structure. We stratified the cohort into two groups by principal component analysis (Additional file 1: Figure S5), and used meta-analysis, which reduced inflation to levels comparable to those from linear mixed model analysis [48] for independent SNPs (Additional file 1: Figure S6). Quantile-quantile plots of pathways under collective inference indicated distributions (Fig. 4f) comparable to those of elevated plus maze for mice (Fig. 4d). Overall, fear of noise, the trait most strongly associated under independent-SNP analyses [5], was also more strongly associated with pathways than was fear of humans/objects.

The pathway most strongly associated with fear of noise (Fig. 5h) was *Hemostasis* (*P* = 7.4×10^−7^), providing further support to the suggested contributions of coagulation factors to cued fear in mice (Fig. 5a) and serotonin in platelets to prepulse inhibition (Fig. 5c). Hemostasis is a large pathway (3592 SNPs) whose association was entirely collective (Additional file 1: Figure S7). Two pathways among those exceeding the Bonferroni threshold for fear of noise were *Reversal of alkylation damage by DNA dioxygenases* (*P* = 1.6×10^−5^) and *LRR FLII-interacting protein 1 activates type I interferon production* (*P* = 1.5×10^−5^). One possible route through which polymorphisms in these DNA damage and innate immune pathways could affect the fear response is via the neural development of cortical interneurons [49], whose disruption can lead to variations in the ability to control fear. Further relevance of these pathway groups to fear response is suggested by recent findings linking stress-hormone action to DNA damage and cytosolic detection of DNA [50, 51]. Pathways highly ranked for fear of humans/objects (Additional file 1: Figure S8) represented a range of similar and other developmentally relevant processes, including apoptosis (*Breakdown of nuclear lamina*, *P* = 1.9×10^−5^). We found that *Synthesis of inositol phosphates (IPs) in the nucleus* was also relatively highly ranked for fear of humans/objects albeit without exceeding the Bonferroni threshold (*P* = 4.9×10^−5^), consistent with the high association between *Effects of PIP2 hydrolysis* and cued fear in mice (Fig. 5a). These pathways highly ranked for fear in dogs were all predominantly collective in nature, with no constituent SNPs dominant in independent-SNP association levels (Additional file 1: Figure S7).

### Narrow- and broad-sense heritability estimates for pathways

**Fig. 8:**
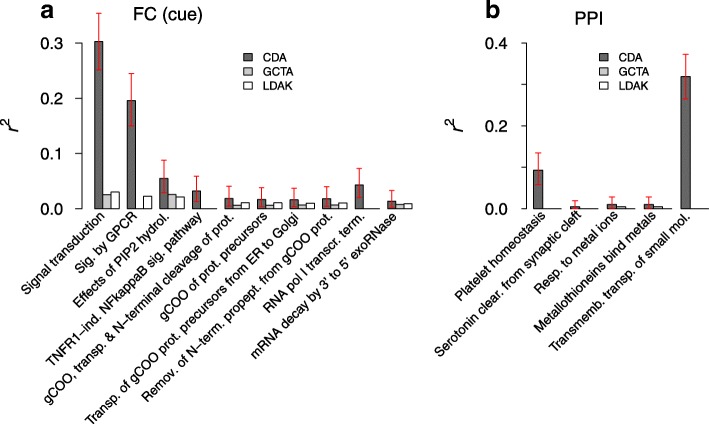
Broad-sense heritability of pathways compared to proportion of additive variance explained. **a** Fear conditioning (cue test) in mice. **b** Prepulse inhibition (PPI) in mice. The top-ranked pathways in Fig. 5a,c are shown in the same order. CDA values represent *r*^*2*^ estimated using regularization conditions determined from cross-validation applied to half of the whole cohort and repeating the inference for the other half. Error bars represent the 95% c.i. The GCTA and LDAK outcomes represent the proportion of variance explained by the same set of SNPs but without interaction effects. For pathways in which the GCTA/LDAK *p*-values were higher than 0.05, the proportion of variance was set to zero. The CDA *p*-values are all smaller than 10^−3^ (Fig. 5)

We estimated the proportion of variance explained by interacting SNPs for a selection of top-ranked pathways associated with mouse and dog behavioral traits (Fig. 8). In contrast to standard linear regression analyses involving low-dimensional predictors, in our approach, the proportion of variance explained *r*^2^ was obtained by evaluating the correlation between predicted and observed phenotypes using the optimal penalizing conditions determined from cross-validation (Fig. 1). This definition also implies that the genetic component of *r*^2^ corresponds to the broad-sense heritability that is, in general, non-additive. We compared our estimates of these heritability estimates for pathways highly ranked for cued fear and prepulse inhibition in mice (Fig. 8a–b) with the additive (narrow-sense) heritability computed by GCTA [33] and LDAK [34]. The narrow-sense heritability values computed by the two methods were similar, with those obtained by LDAK being relatively larger in magnitude overall. In contrast, broad-sense heritability was substantially larger to a varying degree, but typically more so for larger pathways, which hold more room for non-additive effects (Fig. 8a).

## Discussion

We introduced a quantitative trait-mapping approach that targets collective associations of a group of SNPs while taking into account inter-variant interaction effects as well as the effects of non-uniform, empirical high-dimensional distributions of genotypes within the cohort. Performance tests of the algorithm suggested a substantial enhancement in power compared to regression-based methods, similar to the finding for binary phenotypes [10]. Although the approach is marginally more demanding computationally than case-control analyses, the usual advantage of quantitative trait inference, of requiring smaller sample sizes to achieve similar levels of power, is expected to apply under collective inference as well. In addition to the quantitative trait data covered here and the binary case-control data considered in a previous study, one could also analyze categorical data with multiple discrete phenotypes. Categorical data can be treated by an extension of the binary phenotype formulation (Supplementary Text 1 in [10]). A limitation shared by both quantitative and discrete phenotype versions of our method is the reliance on a pathway database, which presumably influences the results strongly.

We demonstrated the practical utility of our approach by applying it to the genotype-phenotype data sets of outbred mice [8] and pet dogs [5]. In contrast to human studies for which reference panels of common variants are available and typical LD values decay rapidly within genomic loci, these early mammalian genomic data still contain much higher degrees of LD, limiting the resolution of standard SNP-based analyses and making the identification of causal genes or SNPs challenging. Our collective inference approach has the potential to reveal groups of variants whose associations with a given trait are non-additive and therefore are relatively insensitive to the spatial extent of fine-scale correlations within a locus. Behavioral traits, for which typically SNP-based inferences yield relatively few dominant associations and whose genetic architectures are often highly polygenic, are especially suited to the approach.

Our inference outcomes for major behavioral traits (Fig. 5) suggest that the nature of genetic associations of a given pathway can span the range between the additive limit, where a few dominant SNPs independently account for the association, to the purely collective limit, where a large number of SNPs spanning multiple loci of negligible individual association levels combine to produce a strong signal. Examples of additive groups are *γ-carboxylation of protein precursors* for cued fear in mice, for which the variants near the *F2* gene had *p*-values close to that of the pathway as a whole (Fig. 6b), *Interconversion of polyamines* and *Hydrolysis of LPC* for anxiety in mice (Fig. 7a), where one or more of the genes located within associated loci likely raised the association strengths of pathways containing them. Examples of pathways with purely collective association for mice are *Effects of PIP2 hydrolysis* (Fig. 6a), *Platelet homeostasis* (Fig. 6d), and *IL-10 signaling* (Fig. 7a), whose associations cannot be reduced to a few SNPs or genes. Notably, genetic factors associated with fear in dogs (Fig. 5h) were all predominantly collective (Additional file 1: Figure S7).

One of our major findings on the genetics of behavioral traits is for fear conditioning in the cued test (Fig. 5a): γ-carboxylated proteases (thrombin coded by *F2*, Fig. 6b) activate neuronal PAR1, triggering G protein-coupled signaling cascades (*Effects of PIP2 hydrolysis*, Fig. 5a) and long-term potentiation. Our previous observation that the same pathway groups were associated with human PTSD [12] provides strong support not only for our current interpretation, but also for the relevance of fear conditioning in mice as a model of PTSD. Bourgognon et al. explicitly demonstrated that this PAR1-G protein coupling activated by thrombin occurs in amygdala neurons, allowing for dynamic modulation of fear in mice [22]; they found that in fear-naive mice, PAR1 couples with Gα_q_ (excitatory) and Gα_o_ (inhibitory) proteins, whereas the latter becomes more important after conditioning. Our finding of the high association of PIP2 hydrolysis, which is downstream of the Gα_q_ protein pathway, suggests that genetic polymorphisms affecting the generation of second messenger molecules during the excitatory phase of long-term potentiation contributes significantly to the heritability of conditioned fear responses.

The role of the thrombin-PAR1 pathway in long-term potentiation and fear conditioning, furthermore, suggests a possible explanation for the commonly observed comorbidity of PTSD and cardiovascular diseases [25, 26]: individuals with collections of genetic polymorphisms that affect this neuronal pathway would also be at higher risk of impaired hemostasis and cardiovascular functions. The *Hemostasis* pathway was also found to be most strongly associated with gene sets differentially expressed in blood from PTSD subjects [52]. A second arm that is likely also contributing to this comorbidity involves the role of serotonin in neuronal functions and psychiatric disorders, including fear conditioning [44, 53] as well as in platelet homeostasis [45]. We found that these overlapping functions of serotonin were associated with prepulse inhibition in mice (Fig. 5c) and further replicated the association between hemostasis and fear from the analysis of the fear of noise in dogs (Fig. 5h). The association of polyamine pathways with the elevated plus maze test (Fig. 5d) is consistent with the known roles of polyamines in anxiety and depression, as for instance demonstrated in studies of high- and low-anxiety mice [54].

The numerous other pathways highly ranked for the behavioral traits of mice and dogs (Fig. 5) belong to classes of processes including cell cycle, axon guidance and migration, DNA repair, innate immune response, apoptosis, and cellular stress response. Together, they are consistent with the view that individual variation in behavioral traits are strongly affected by disruptions to neurodevelopmental processes, owing to the collective effects of polymorphisms, which likely result in impaired development of key neuronal structures such as cortical interneurons [12, 49]. The strong associations of DNA repair and type I interferon-mediated immune response pathways with fear of noise in dogs (Fig. 5h), furthermore, support recent experimental findings suggesting that stress is linked to inflammation via DNA damage and the resulting recognition of damaged DNA in the cytosol [50, 51].

Although the genetic architectures of mice/dogs differ markedly from those of humans, the overall picture suggested by our results on fear-related traits is likely to be relevant to human genetics, given the common evolutionary origin of fear responses shared by all mammals. Results using animal genetic data may also offer avenues for experimental validation. For instance, pharmacological experiments that target neuronal pathways involving γ-carboxylated proteases in mice [22] could benefit from the genetic screening results in this work, and may help identify similar drug candidates for treating human PTSD and other psychiatric conditions.

Our estimates of the variance explained by interacting SNPs (Fig. 8) demonstrate that broad-sense heritability can be computed from genomic data for unrelated individuals. Furthermore, extensive epistatic effects among many SNPs make the heritability of different pathways non-additive.

## Conclusions

We presented a novel method to infer collective association of a large number of variants with quantitative traits while taking into account interaction effects. Applications to mammalian behavioral trait data revealed pathways linking stress-related phenotypes and hemostasis: neuronal signaling by γ-carboxylated proteases. Our work provides evidence suggesting that behavioral traits are strongly influenced by large-scale interaction effects among genetic variants.

## Additional files

Additional file 1: **Table S1.** Sample sizes of behavioral trait data considered for outbred mice^8^ and dogs^5^. **Figure S1** Distributions of quantitative trait values used for association testing. See **Table S1**. c, e, h, and i have been log-transformed (c and e from percentages, and h and i from a scale of 1 to 5). **Figure S2** Comparison of single-SNP *p*-values from linear regression (LR) and continuous discriminant analysis (CDA). **Figure S3** Comparison of empirical *p*-values of groups of interacting SNPs estimated by permutation of phenotype labels (symbols) and the use of the null distribution of R for normally distributed data. **Figure S4** Optimized prediction scores of 10 top-ranked pathways for fear conditioning (FC; cued test). **Figure S5** Population stratification of Labrador Retrievers using principal component (PC) analysis. **Figure S6** Quantile-quantile plots of independent SNP *p*-values for Labrador Retriever data. **Figure S7** Independent-SNP and collective association levels of SNPs in pathways highly ranked for fear in dogs. **Figure S8** Top-ranked pathways for fear of humans/objects (dogs). **Table S2** Additional pathways from Reactome database (Feb. 2018) ranked by association strengths with respect to fear conditioning (cued test). (PDF 1811 kb)

Additional file 2: **Table S3.** Lists of highly ranked pathways for mouse and dog behavioral traits. Pathways with *P* < 0.05 are shown for fear conditioning (cued and context), prepulse inhibition, elevated plus maze, swim test, sleep duration and difference in light and dark periods, noise and human/object-oriented fear in dogs. (XLSX 120 kb)

## Abbreviations

CDA: Continuous discriminant analysis
DAG: Diacylglycerol
ER: Endoplasmic reticulum
FDR: False discovery rate
GPCR: G protein-coupled receptor
IP: Inositol, phosphate
IP3: Inositol 1,4,5-triphosphate
LD: Linkage disequilibrium
MAPK: Mitogen-activated protein kinase
PAR1: Protease-activated receptor 1
PIP2: Phosphatidylinositol 4,5-phosphate
PL: Pseudo-likelihood
PTSD: Post-traumatic stress disorder
RR: Ridge regression
SNP: Single-nucleotide polymorphism
