# Supplementary material for: Collective interaction effects associated with mammalian behavioral traits reveal genetic factors connecting fear and hemostasis
Source: BMC Psychiatry. 2018 Jun 5;18:175. doi: 10.1186/s12888-018-1753-4 (PMC5989392; doi:10.1186/s12888-018-1753-4)
Supplement: Supplementary file 1 — Table S1. Sample sizes of behavioral trait data considered for outbred mice8 and dogs5. Figure S1 Distributions of quantitative trait values used for association testing. See Table S1. c, e, h, and i have been log-transformed (c and e from percentages, and h and i from a scale of 1 to 5). Figure S2 Comparison of single-SNP p-values from linear regression (LR) and continuous discriminant analysis (CDA). Figure S3 Comparison of empirical p-values of groups of interacting SNPs estimated by permutation of phenotype labels (symbols) and the use of the null distribution of R for normally distributed data. Figure S4 Optimized prediction scores of 10 top-ranked pathways for fear conditioning (FC; cued test). Figure S5 Population stratification of Labrador Retrievers using principal component (PC) analysis. Figure S6 Quantile-quantile plots of independent SNP p-values for Labrador Retriever data. Figure S7 Independent-SNP and collective association levels of SNPs in pathways highly ranked for fear in dogs. Figure S8 Top-ranked pathways for fear of humans/objects (dogs). Table S2 Additional pathways from Reactome database (Feb. 2018) ranked by association strengths with respect to fear conditioning (cued test). (PDF 1811 kb) [file 12888_2018_1753_MOESM1_ESM.pdf]

Collective interaction effects associated with mammalian behavioral traits reveal genetic factors connecting fear and hemostasis: Supplementary Information

H. J. Woo & J. Reifman

Table S1: Sample sizes of behavioral trait data considered for outbred mice[8] and dogs[5]. Variables refer to names in the original reports. Fractional quantities were log-transformed before use with small constants added to avoid singular values. Covariates are variables used to stratify the cohort into two sub-groups for meta-analysis. EPM, elevated plus maze; FC, fear conditioning; PC, principal component; PPI, prepulse inhibition.

		Variable	Covariate	n
FC	Cue	FC.Cue.MeanFreeze.Corrected	Sex	1,716
	Context	FC.Context.Freeze.Corrected	Sex	1,667
PPI		SPPI.pc_average_ABC	Sex	1,572
EPM		Open = EPM.OpenArms.Time<br>Closed = EPM.ClosedArms.time<br>Closed/ (Open + Closed)	Sex	1,592
Forced swim		PST.Immobility.Last4min	PST.Immobility.First2min	1,659
Sleep	Duration	Sleep.s24h	Sex	1,448
	Difference (light/dark)	Sleep.sDif_LD	Weight.Average	1,065
Fear (dogs)	Noise	NoiseFear	PC1	868
	Human/object	HOFear	PC1	882

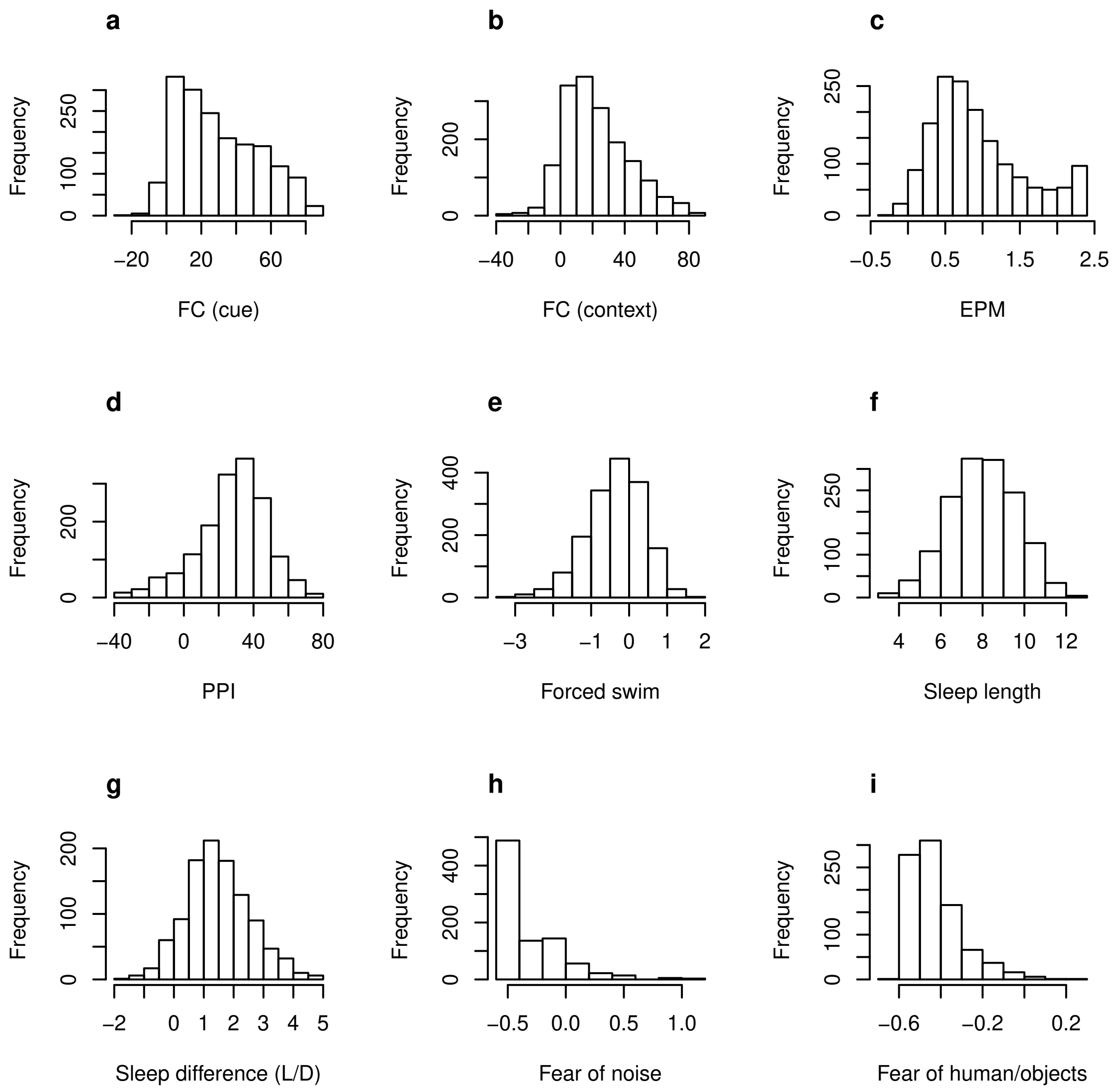

Figure S1: Distributions of quantitative trait values used for association testing. See Table S1. **c**, **e**, **h**, and **i** have been log-transformed (**c** and **e** from percentages, and **h** and **i** from a scale of 1 to 5).

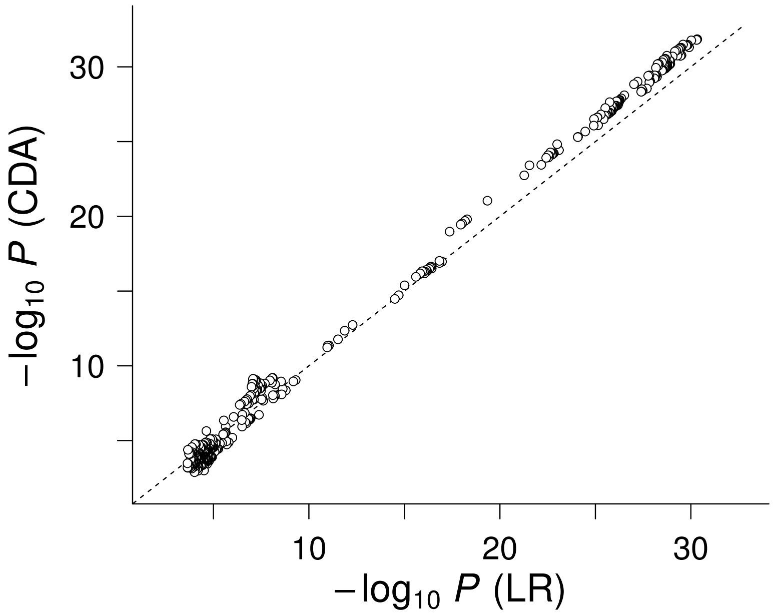

Figure S2: Comparison of single-SNP p-values from linear regression (LR) and continuous discriminant analysis (CDA). The genotype-phenotype combination of mouse data for bone mineral content ("BMC.mean") was used to obtain LR p-values for all SNPs. SNPs with LR $P < 1 \times 10^{-3}$ were then selected (622 in total) and CDA inference was performed for each SNP separately ($m = 1$) under 5-fold cross-validation. The prediction scores R were then transformed into p-values using the t-distribution as the null distribution. The dotted line is the diagonal.

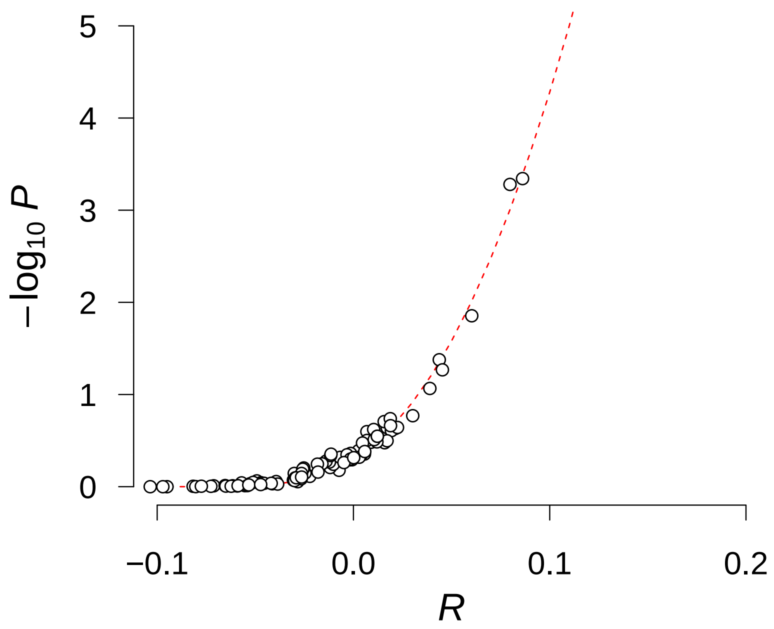

Figure S3: Comparison of empirical p-values of groups of interacting SNPs estimated by permutation of phenotype labels (symbols) and the use of the null distribution of R for normally distributed data. The latter was obtained from $t > R\sqrt{(n-2)/(1-R^2)}$ in the t-distribution with d.f. $= n-2$, which was indistinguishable from $P = 1 - \Phi(z)$ where Φ is the c.d.f. of the standard normal distribution and $z = (1/2)\sqrt{n-3}\ln\left[(1+R)/(1-R)\right]$. $n = 1{,}716$ is the sample size. The SNP groups for genes were formed by selecting variants (CFW mice) within 50 kb of the coding regions. Continuous discriminant analysis inference was first performed for the association with fear conditioning (cued test) traits for each SNP group under the alternative hypothesis. The inference was then repeated under the null hypothesis with phenotype-label permutation for $\sim 1{,}000$ replicates. The p-value was estimated as the fraction of times the null R was higher than the alternative R.

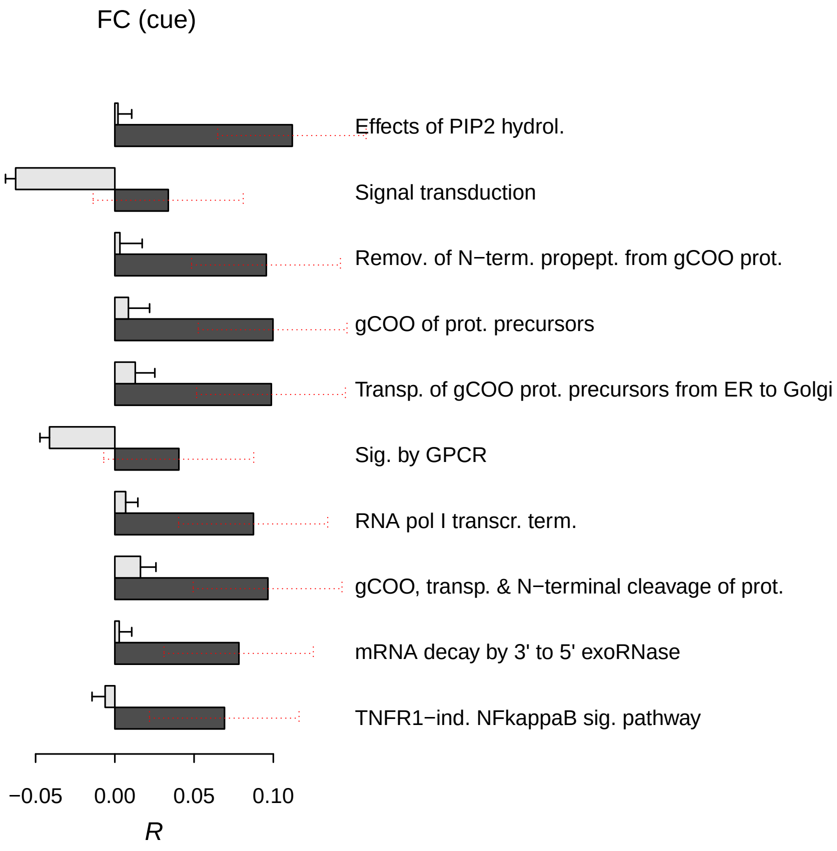

Figure S4: Optimized prediction scores of 10 top-ranked pathways for fear conditioning (FC; cued test). Bars in dark and light colors represent the values of R (alternative hypothesis) and the mean of R_0 (null hypothesis sampled with phenotype-label permutation). The error bars on R and R_0 represent the 95% c.i. and the standard error of means ($n = 10$), respectively. ER, endoplasmic reticulum; gCOO, γ-carboxylation; GPCR, G protein-coupled receptor; hydrol., hydrolysis; ind., induced; PIP2, phosphatidylinositol phosphate 2; pol I, polymerase I; propept., propeptide; prot., protein; remov., removal; sig., signaling; term., termination; transcr., transcription; transp., transport.

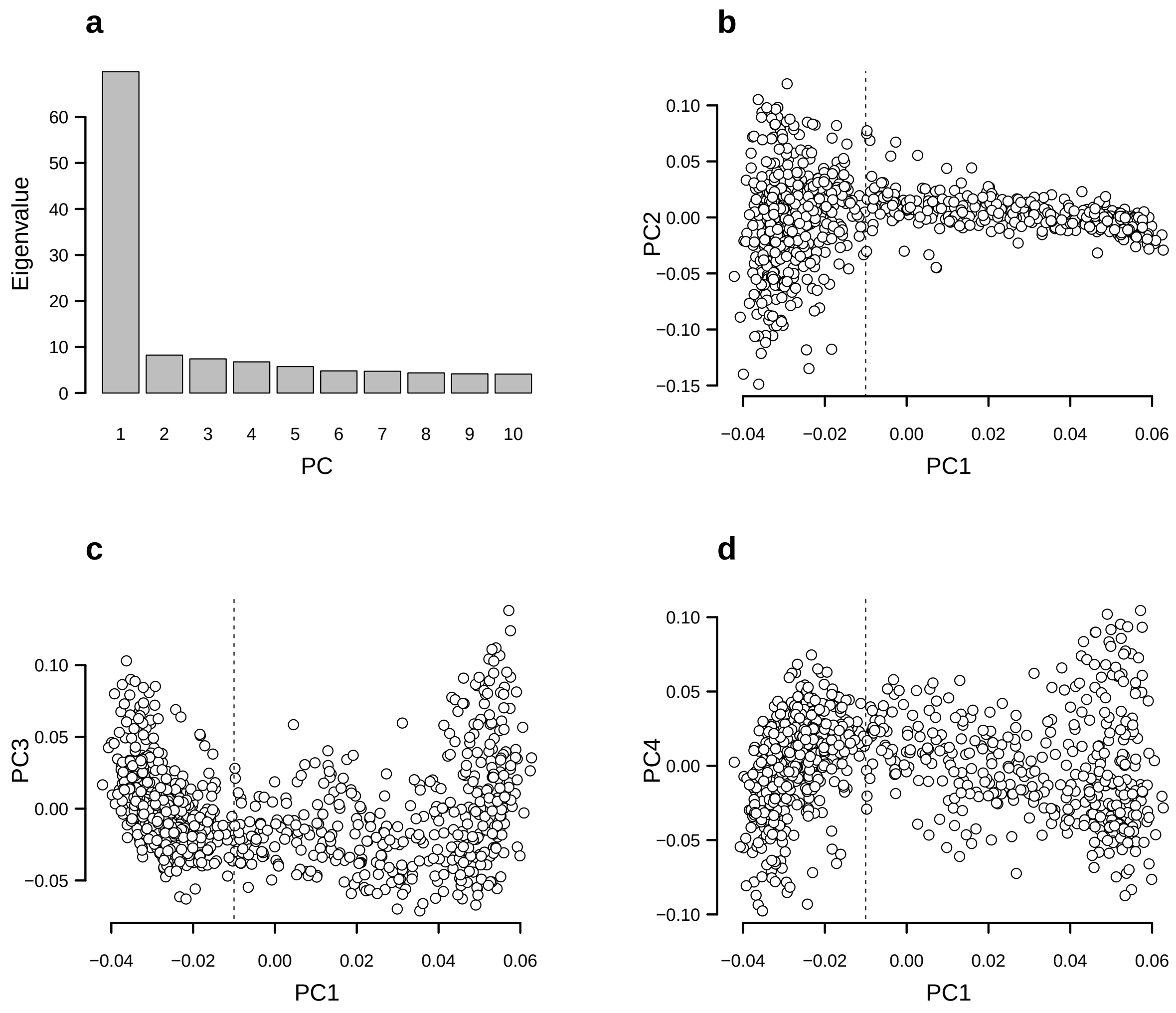

Figure S5: Population stratification of Labrador Retrievers using principal component (PC) analysis. Results for the human/object-oriented fear trait are shown ($n = 882$). (**a**) Top ten PC eigenvalues. (**b-d**) Scatterplots of PC1 versus PC2-4. The vertical dotted lines repesent the cutoff we used for PC1(-0.01).

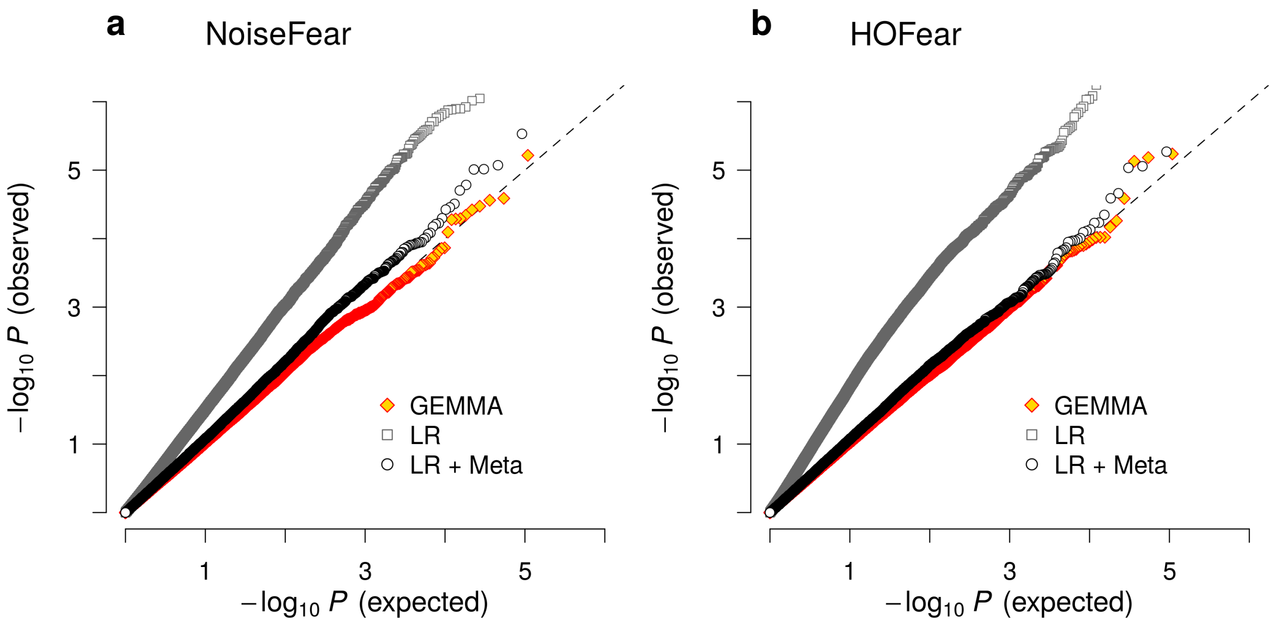

Figure S6: Quantile-quantile plots of independent SNP p-values for Labrador Retriever data. (**a**) Fear of noise association. (**b**) Human/object fear association. Results from linear regression applied to the whole cohort ("LR") and from meta-analysis ("LR+Meta") with two sub-groups stratified by the first principal component (see Fig. S5) are compared with those from GEMMA[48].

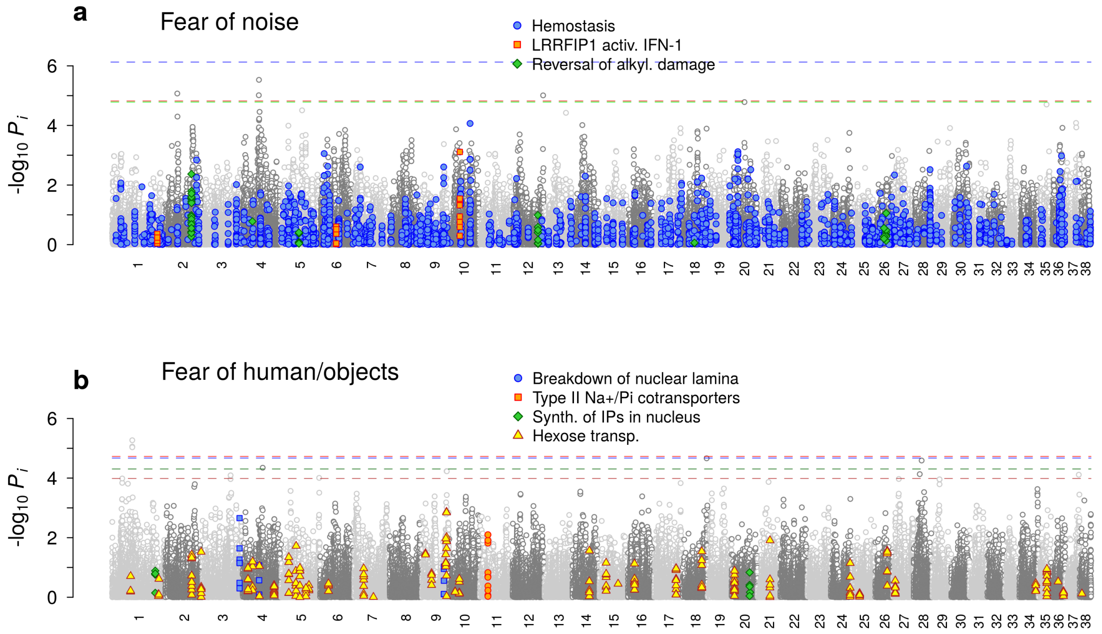

Figure S7: Independent-SNP and collective association of SNPs in pathways highly ranked for fear in dogs. (**a**) Fear of noise and three top-ranked pathways: *Hemostasis*, *LRRFIP1 activates type I interferon production*, and *Reversal of alkylation damage by DNA dioxygenases*. (**b**) Fear of humans/objects and four top-ranked pathways: *Breakdown of nuclear lamina*, *Type II* Na^{+}*/Pi cotransporters*, *Synthesis of inositol phosphates in nucleus*, and *Hexose transport*. The dotted horizontal lines represent the collective inference p-values of the pathways. Activ., activates; alkyl., alkylation; IFN, interferon; IP, inositol phosphate; LRRFIP1, leucine-rich repeat flightless-interacting protein 1; Pi, phosphate; synth., synthesis; transp., transport.

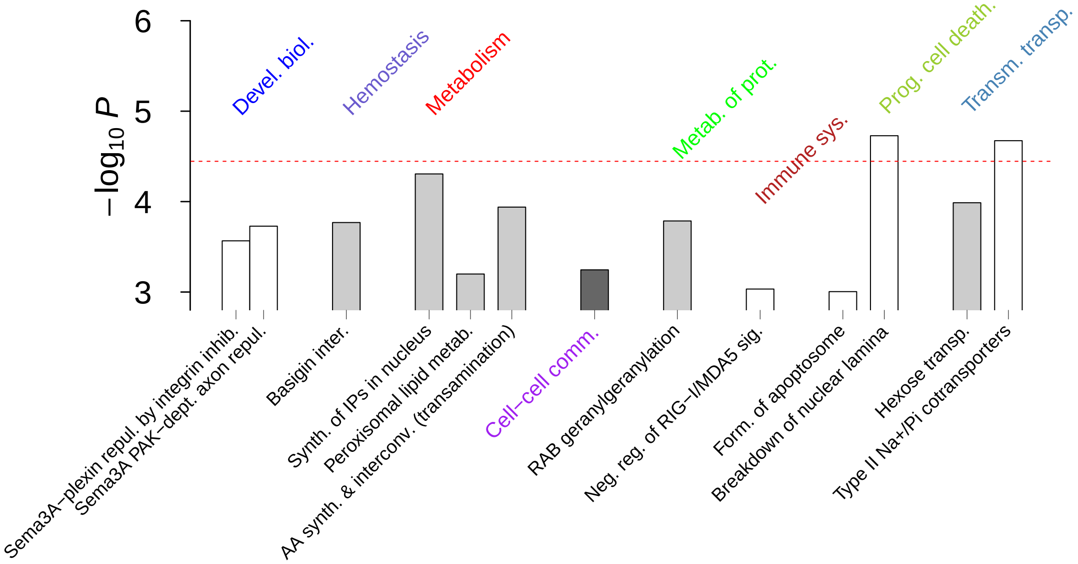

Figure S8: Top-ranked pathways for fear of humans/objects (dogs). See Fig. 5f for the quantile-quantile plot. The dotted line represents the Bonferroni-corrected threshold. AA, amino acid; comm., communication; dept., dependent; devel. biol., developmental biology; form., formation; inhib., inhibition; inter., interaction; interconv., interconversion; IP, inositol phosphate; MDA5, melanoma differentiation-associated protein 5; metab., metabolism; neg., negative; PAK, p21 activated enzyme; Pi, phosphate; prog., programmed; reg., regulation; repul., repulsion; RIG-I, retinoic acid-inducible gene I; Sema, semaphorin; sig., signaling; synth., synthesis; sys., system; transp., transport.

Table S2: Additional pathways from Reactome database (Feb. 2018) ranked by association strengths with respect to fear conditioning (cued test). Pathways with $P \leq 0.05$ among those not in the main database (Fig. 5) are shown. CREB, cyclic AMP response element-binding protein; IL, interleukin; RUNX, Runt-related transcription factor; SDK, Sidekick; sig., signaling; transcr., transcription.

Pathway	No. of SNPs	R	R_0	P
IL-33 sig.	24	0.037	-0.0246	3.8×10^{-3}
CREB3 factors activate genes	73	0.063	0.0022	4.2×10^{-3}
RUNX1 regulates transcr. of genes involved in IL sig.	35	0.058	0.0021	7.6×10^{-3}
SDK interactions	13	0.041	-0.0026	2.9×10^{-2}
IL-21 sig.	172	0.037	-0.0007	5.0×10^{-2}
